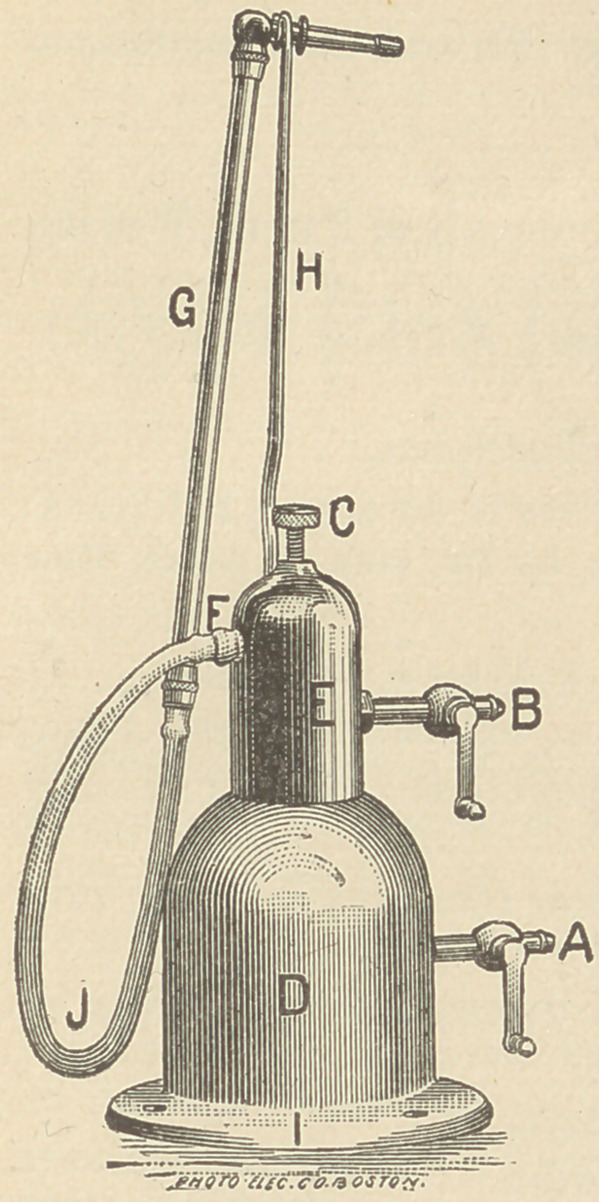# Current News and Opinions

**Published:** 1888-12

**Authors:** 


### Current News.

### DOMESTIC CORRESPONDENCE.

To the Editor:

The chief cause of failures, when moulding celluloid by either steam, oil or dry heat, is owing to the form of the blanks, which could be easily remedied by changing the moulds; which necessitates from a half to an hour's hard work of scraping and fileing, with the result then of only a faint approximation of what a blank should be.

That the color of the celluloid blanks could be made to resemble every shade of mucous membrane, no one can doubt who is at all familiar with the innumerable articles of every conceivable shade now on the market.

The tendency of celluloid to warp and discolor can be overcome by moulding it in a plaster investment heated to a temperature of 315° in a dry chamber.

The moulding at this high degree of heat must be done inside of five minutes, or the celluloid will either become porous or burn.

The judgment now necessary to mould a celluloid plate in five minutes, and have the denture come out perfectly finished, all ready to be placed in the patient's mouth, without any fileing or polishing, requires mechanical ability of a high order trained by long experience.

It seems to me that the following method will enable any mechanical dentist to make a perfect celluloid dentine every time without a failure:

Set the teeth up in paraffine in the usual manner, trim and shape the paraffine to the exact form you wish the celluloid plate to have, run a small bead of paraffine around over the pins, dip the case in cold water and remove the teeth with the thumb and finger; partially fill the cavities left by the teeth with melted paraffine, invest this paraffine pattern on a tin die in plaster, the same as you would if the teeth were in place.

Scrape and file a celluloid blank, especially in the roof, until it is as near the form of the paraffine pattern as possible. Heat the plaster investment, and mould at about 270°. Get the plaster all off of the celluloid, even if you have to polish it. You will then have a *perfect blank*, which can be easily moulded into the teeth in five minutes, without any danger of crushing the teeth or cracking the investment. This process saves time and teeth, and there is a greater demand for these perfect celluloid plates at $30 each, than for rubber plates at $10 each, by the same class of people.

In the six years that I have made celluloid plates by the dry heat process, I have never seen a broken one. The teeth break the same as on other bases.

To replace a broken tooth: Select a tooth, cover the pins with an excess of paraffine, place it paraffine up in the lower half of a flask filled with plaster; fill the upper half of the flask with plaster, open the flask and remove the paraffine; heat the investment and melt a piece of celluloid on to the tooth the same as if you were moulding a whole denture.

Enlarge the cavity, left by the broken tooth, on the lingual side. After grinding the tooth and shaping the celluloid until it fits perfectly, cement it in place with collodion. The union of the celluloid will be perfect, and the tooth will be held as firmly in place as any tooth in the plate. The difficulties encountered in trying to overcome these two points have confined the manufacture of celluloid dentures to a very few dentists.

FREDERICK W. SEABURY.

Providence, R. I., Nov. 17, 1888.

---

TO THE EDITOR:

The third annual meeting of the Western Illinois Dental Society was held at Kewanee, Oct. 23d and 24th. F. Christianer, of Abingdon, the president, called the meeting to order, and, after preliminary business, read the annual address. The afternoon of the 23d and forenoon of the 24th was devoted to clinics. Bushnell was selected as the next place of meeting, and the following officers elected for the ensuing year: J. A. W. Davis, Galesburgh, President; E. M. Robbins, Carthage, Vice-President; A. H. McCandless, Rock Island, Secretary; W. W. Hart, Quincy, Treasurer; J. W. Murphy, Bushell; L. W. Skidmore, Moline; and R. W. Sharp, Kewanee, Executive Committee. The members of the society were given a banquet by the Kewanee dentists and physicians Tuesday evening. These meetings are especially interesting to the younger members of the society, as they are more free to take an active part in the discussion of papers, the giving of clinics, etc., than in the State Society. It is hoped the membership will be largely increased at next meeting. The number of members at present is forty-two.

A. H. MCCANDLESS.

Rock Island, Ill., Nov. 15, 1888.

---

WE have personally investigated the merits of the office coat advertised by Hirsh, Frank & Co., in this issue for the first time. They are neat, well made, and as serviceable a coat as any one need want, and the price, $1.25 each, is remarkably low. When in practice we used to have to pay $4.00 each for office coats.

---

THE art of making stained-glass windows, which had its renaissance in this country within the last twelve years will be the subject of a popular and appreciative paper in the Christmas number of *Scribner's Magazine*, by Will H. Low, the artist, whose illustrations of Keats's poems have been so much praised. Some of the best work of John La Farge, Louis C. Tiffany, Francis Lathrop, Lyell Carr, and others will be reproduced among the illustrations.

---

We desire to call attention to the advertisement on page 22. We have used the brush there mentioned and find it the most convenient little brush on the market for cleansing the inner surfaces of the teeth and around third molars.

The articular has long been in use, and needs no word of praise from us. It is an instrument based upon scientific principles. If you really desire to articulate a set of teeth, scientifically, get one.

### A NEW NITROUS-OXIDE BLOWPIPE.

At the Union meeting, Boston, July, 1888, Dr. G. F. Harwood, of Worcester, Mass., presented to the profession without let or hindrance as regards its manufacture or sale the following described Nitrous Oxide Blow-Pipe.

The apparatus is designed to take Nitrous Oxide from the ordinary gasometer, at low pressure, or from the cylinder at high pressure, and economically combine the same with ordinary illuminating gas in any desired proportion for soldering gold or platinum, melting at high temperatures, or for any purpose where the perfect regulation and control of a powerful and concentrated flame is required.

It consists of an expansion chamber or reservoir *D*, provided with a lever stop-cock *A*, which is to be connected with the Nitrous Oxide supply at the gasometer or cylinder by strong rubber tubing. Above this is the mixing chamber *E*, having a lever stop-cock *B*, connected by rubber tubing with the illuminating gas supply.

The chambers *D*, and *E*, are separated by a diaphragm having a regulating valve *C*, which perfectly controls the admixture of the two gases, which, when combined, are conducted through the outlet *F*, and flexible tubing *J*, to the blow-pipe tube *G*. This is provided with two interchangeable nozzles with which to secure a large or small flame, a curved wire standard *H* furnishes support for the nozzle when not in use.

The flange *I* is drilled for screws by which the apparatus may be secured in any convenient position to the bench, shelf or wall.

A stand for holding the N. O. Cylinder and blow-pipe apparatus on one base while not strictly necessary, is recommended for convenience, and will be furnished when desired.

Extra heavy cloth-lined rubber tubing for connecting with cylinder and reducing couplings connecting a large tube to a small one will be furnished to order.

The apparatus is neat and complete in every respect, and will take gas from either cylinder or gasometer, a point that is not covered by any other Nitrous Oxide Blow-Pipe on the market. We take pleasure in recommending it to the profession.

---

WE desire to call attention to the advertisement of George E. Hodge in this issue. His stone cut burs are generally known and universally commended by all who use them.

### ODONTOLOGICAL SOCIETY OF PENNSYLVANIA—TENTH ANNIVERSARY MEETING, ASSOCIATION HALL, CORNER FIFTEENTH AND CHESTNUT STREETS, PHILADELPHIA, DEC. 12-13, 1888.

Reduced rates have been secured at the Colonade, 15th and Chestnut Streets, which will be made headquarters.

PROGRAM:

Call to order by the President, Dr. E. C. Kirk, promptly at 2 P. M., Wednesday, December 12.

Exercises will be opened by prayer by Rev. Wayland Hoyt, D.D., Philadelphia.

Introductory address by Prof. Chas. J. Essig, Philadelphia.

Response by Dr. A. L. Northrop, New York, followed by papers as follows:

Etiology of Caries, A Few Thoughts Thereon, by Dr. Geo. S. Allan, New York.

Removable Crown and Bridge Work, by Dr. S. S. Waters, Baltimore.

The evening session will be given to Lantern exhibits by Drs. Allan, Andrews and Sudduth, illustrating their views of Dental Histology and Pathology in their direct bearing upon the problem of decay.

Thursday morning will be devoted to clinics at the Hazeltine building, 1416 Chestnut Street.

Dr. Edwin P. Wright, Richmond, Va., will demonstrate his method of bleaching teeth.

Dr. W. Storer How, Philadelphia, Porcelain Inlays.

Dr. H. A. Parr, New York, Removable Bridge-work.

Dr. H. C. Register, Philadelphia, subject to be announced.

Dr. W. G. A. Bonwell, Philadelphia, will exhibit his system of correctors, with remarks and demonstrations from practical cases.

Dr. A. G. Bennett, Philadelphia, Bridge-work.

Dr. J. A. Woodward, Philadelphia, will show his illuminating apparatus.

Dr. E. P. McLean, Boston, will give a clinic on his method of finishing fillings, sharpening and polishing instruments.

Dr. T. S. Waters, Baltimore, will show Crown and Removable Bridge-work.

Dr. H. W. F. Buttner, Baltimore, will demonstrate his method of mounting Crown and Bridge-work, with instruments used.

Thursday afternoon, 2 P. M., Association Hall, Dr. James Truman, Philadelphia, will read a paper on Treatment and filling of root canals.

Dr. S. H. Guilford, Philadelphia, will give a paper on The Voluntary Movement of Teeth, Causing Abnormal Interdental Spaces.

Dr. S. G. Perry, New York, will treat of The treatment of proximate surfaces.

L. ASHLEY FAUGHT, D.D.S., Chairman.

DANIEL NEAL MCQUILLAN, D.D.S.,

Secretary of the Anniversary Committee.

## Figures and Tables

**Figure f1:**